# Spatial model for risk prediction and sub-national prioritization to aid poliovirus eradication in Pakistan

**DOI:** 10.1186/s12916-017-0941-2

**Published:** 2017-10-11

**Authors:** Laina D. Mercer, Rana M. Safdar, Jamal Ahmed, Abdirahman Mahamud, M. Muzaffar Khan, Sue Gerber, Aiden O’Leary, Mike Ryan, Frank Salet, Steve J. Kroiss, Hil Lyons, Alexander Upfill-Brown, Guillaume Chabot-Couture

**Affiliations:** 1 Institute for Disease Modeling, 3150 138th Ave SE, Bellevue, WA 98005 USA; 2 National Emergency Operations Centre for Polio Eradication, Islamabad, Pakistan; 3 World Health Organization, Islamabad, Pakistan; 4 Bill and Melinda Gates Foundation, Seattle, WA USA; 5 United Nations Children’s Fund (UNICEF), Islamabad, Pakistan; 6 David Geffen School of Medicine, University of California, Los Angeles, CA USA

**Keywords:** Disease mapping, Polio eradication, Risk mapping, Spatial epidemiology, Hurdle models, Pakistan, Risk prioritization, Vaccination campaigns, Supplementary immunization activities

## Abstract

**Background:**

Pakistan is one of only three countries where poliovirus circulation remains endemic. For the Pakistan Polio Eradication Program, identifying high risk districts is essential to target interventions and allocate limited resources.

**Methods:**

Using a hierarchical Bayesian framework we developed a spatial Poisson hurdle model to jointly model the probability of one or more paralytic polio cases, and the number of cases that would be detected in the event of an outbreak. Rates of underimmunization, routine immunization, and population immunity, as well as seasonality and a history of cases were used to project future risk of cases.

**Results:**

The expected number of cases in each district in a 6-month period was predicted using indicators from the previous 6-months and the estimated coefficients from the model. The model achieves an average of 90% predictive accuracy as measured by area under the receiver operating characteristic (ROC) curve, for the past 3 years of cases.

**Conclusions:**

The risk of poliovirus has decreased dramatically in many of the key reservoir areas in Pakistan. The results of this model have been used to prioritize sub-national areas in Pakistan to receive additional immunization activities, additional monitoring, or other special interventions.

**Electronic supplementary material:**

The online version of this article (doi:10.1186/s12916-017-0941-2) contains supplementary material, which is available to authorized users.

## Background

The Global Polio Eradication Initiative (GPEI) has seen great success since its launch in 1988. At the time of this writing, only Pakistan, Afghanistan, and Nigeria remain endemic for polio with only 37 cases of wild poliovirus serotype 1 (WPV1) recorded in 2016. The 20 cases reported in Pakistan in 2016 represent a historically low case count for a calendar year and a 63% reduction in cases compared to 2015. However, transmission is still occurring on a considerable geographic scale, with four of eight provinces reporting WPV1 cases in 2016. Pakistan has approximately 25 million children under the age of 5 years [1], which presents challenges for allocating limited resources. As the program approaches the goal of zero cases, identifying the districts that are most likely to be infected and prioritizing those districts for interventions is a priority of the program and should accelerate the path towards eradication.

In the Eastern Mediterranean Region of the World Health Organization (WHO) key strategies of the of GPEI included (1) achieving high coverage of at least three doses of oral polio vaccine (OPV), (2) implementation of supplementary immunization activities (SIAs), and (3) the development of sensitive epidemiological and laboratory surveillance using standard WHO definitions [2]. Eradication efforts began in Pakistan in 1994, when the first SIA was conducted, and in 1995, when acute flaccid paralysis (AFP) surveillance commenced [3]. The case burden in Pakistan has dramatically decreased since the 1990s, but sustained implementation of the first two GPEI strategies has been challenging due to poor rates of routine immunization (RI) and security issues. As of 2012, only 53% of children in Pakistan were receiving all basic vaccines, including Bacillus Calmette–Guerin, measles and three doses of polio and diphtheria, pertussis, and tetanus, with provincial rates as low as 16% and 29% in Balochistan and Sindh provinces, respectively [4]. Additionally, vaccination bans and security limitations in the Federally Administered Tribal Areas (FATA) and Khyber Pakhtunkhwa (KP) and violence against vaccinators in FATA, Balochistan, and Sindh provinces have periodically limited the program’s efforts to consistently implement SIAs with high population coverage in conflict-affected areas since 2008 [5]. These programmatic challenges have resulted in pockets of underimmunized (fewer than three OPV doses) children and have allowed transmission to persist.

Since 2011, the Pakistan program has been implementing and enhancing a National Emergency Action Plan (NEAP) for polio eradication to improve management and accountability strategies, highlight core reservoirs of transmission, and to ensure the program is creating and using high quality data [5, 6]. To aid in the prioritization of sub-national areas for programmatic interventions, we developed a spatial model to estimate the risk of future WPV1 cases for the 155 districts of Pakistan. Previous studies highlight the utility of spatial risk models for guiding programmatic interventions for polio, such as the 86% accuracy for predicting districts at risk for future WPV1 cases in Nigeria [7]. These models guided the prioritization of sub-national areas for immunization planning and allocation of technical and administrative field personnel. The use of a spatial risk model, which is statistically evaluated based on its accuracy for predicting locations of cases, represents a methodological departure from the common approach of compiling programmatic indicators of disease risk, assigning weights based on expert opinion, and linearly combining into a risk score [8, 9]. Unfortunately, a spatial risk model has not previously been applied to model the risk of WPV1 in Pakistan. In this paper, we will describe our efforts to model the risk of future WPV1 cases in districts of Pakistan and describe how these efforts have been incorporated by the National Emergency Operating Centre (N-EOC) in Islamabad to the 2016–2017 NEAP to prioritize the districts of Pakistan.

## Methods

### Description of data

Our modeling efforts rely on the Pakistan AFP surveillance data, which is managed by WHO and is the source for information on paralytic cases of polio. AFP cases are identified through the extensive surveillance network in Pakistan, which relies on both passive and active (sweeps and case search) surveillance with oversite at the district, provincial, and national level [6]. However, AFP can be caused by many viruses other than WPV [10]. For each childhood AFP case, the initial investigation includes two stool samples (to be tested for poliovirus), demographics, date of paralysis onset, history of OPV through RI as well as SIAs, and clinical symptoms. For the purposes of our analyses, AFP cases that are found to be positive for WPV1 are treated as the outcome of interest and AFP found not to be infected with polio, non-polio AFP (NPAFP), are treated as a random sample from the population [11–13]. A surveillance system that achieves an annual rate of 2 or more NPAFP per 100,000 children under age 15 is considered sufficient to detect circulating WPV1 [14].

### Statistical methods

Using the NPAFP dose histories we constructed estimates of district-level vaccination rates, such as zero-dose RI (received zero OPV doses from RI) and underimmunization (defined as children who have received three or fewer doses of OPV from birth until AFP investigation). However, the number of NPAFP observations in each district within a 6-month period can be quite small, ranging from 0 to 74 in our data, resulting in differences during a 6-month period that are implausible when considering children under 5 years of age. To alleviate this problem, we used a hierarchical Bayesian space–time model with a temporally structured space–time interaction [15] to generate smoothed estimates of district-level vaccination rates. Briefly, the space–time model borrows information over space and time to estimate the underlying rates from which the observed data was drawn. Additional details about the model specifications and interaction selection can be found in Section 1 of Additional file 1.

A dynamic immunity model, originally developed for Northern Nigeria [16], was implemented using Pakistan-specific vaccine efficacies [13] to estimate district-level population immunity. The immunity model uses a hierarchical Bayesian model to estimate district-level, annual, age-specific SIA coverage using the ages and SIA doses reported by NPAFP cases. A hypothetical cohort then progresses through the true SIA calendar experiencing the estimated yearly age-specific SIA coverage rates and vaccine type. The result is monthly district-level serotype-specific estimates of under-five population immunity which have been averaged over each 6-month period.

Polio incidence has been rare and spatially heterogeneous in Pakistan since 2003, with 91% (3804 out of 4185) of 6-month district-level observations reporting zero WPV1 cases. To account for this large number of zeros we implemented a spatial Poisson hurdle model [17] using a hierarchical Bayesian framework. The Poisson hurdle model explicitly models the excess observations of zero cases we would expect from areas without circulating virus with a Bernoulli component as well as the total number of cases given at least one case with a truncated Poisson. The probability of at least one WPV1 case (Bernoulli) and the total number of WPV1 cases given at least one case (truncated Poisson) in a district during a 6-month period were jointly modeled as a function of covariates from the previous period, namely a set of independent and spatially structured random effects, also known as the convolution model [18], and an observation-level random effect to account for overdispersion. Notably, a bivariate prior distribution was assigned to the independent district random effects to allow for a correlation between the district-level Bernoulli and truncated Poisson models. The expected number of WPV1 cases, which is defined as the product of the probability of at least one WPV1 case and the expected number of WPV1 cases given at least one case, was used as the measure of risk. A similar modeling approach has been used to predict WPV1 and WPV3 in Nigeria [7]. Full modeling details, including prior distribution specifications, can be found in Section 2 of Additional file 1.

Model selection was carried out in two stages. In the first stage, we fit models based on the 64 combinations of seasonality (low season is January through June), vaccine-derived immunity for type 1 poliovirus, underimmunized fraction, zero dose RI fraction, recent neighboring cases (defined as paralytic WPV1 cases in neighboring areas in the previous 6 months and square root transformed), and recent cases (defined as paralytic WPV1 cases and square root transformed) for the Bernoulli portion, with only random effects in the truncated Poisson model, and selected the model that minimized the deviance information criteria (DIC) [19]. In the second stage, we included the selected covariates for the Bernoulli portion and considered all combinations of covariates for the truncated Poisson portion of the model and then again selected the covariates which minimized the DIC.

The predictive accuracy of our model was assessed by comparing our predictions to held out data. The selected model was first fit using data from 2003 until the first 6 months of 2009 and then used to predict cases in the second half of 2009. This procedure was repeated, using all historical data, for each subsequent 6-month period with the final predictions for the second half of 2016 relying on data from 2003 through the first 6 months of 2016. For each set of predictions, the area under the curve (AUC) for the receiver operator characteristic (ROC) curve was calculated. The AUC for the ROC curve is a single value that summarizes the shape and position of the ROC curve and has the useful interpretation as the probability that a randomly selected district with a case will have a higher risk score than a randomly selected district without a case [20, 21]. AUCs between 0.7 and 0.9 suggest a moderate predictive power and AUCs above 0.9 suggest a strong predictive power [22].

All modeling was completed in R 3.2.1 [23]. The SIA coverage models were fitted via Markov chain Monte Carlo using RStan [24, 25]. The space–time models used for smoothing vaccination rates and the spatial Poisson hurdle model were fit using the Integrated Nested Laplace Approximation (INLA) [26, 27] as implemented in the INLA package [28, 29], which provides a fast and accurate alternative to Markov chain Monte Carlo methods for these type of space–time models [15, 30–32].

## Results

### Estimated dose history and vaccine-derived immunity

**Fig. 1:**
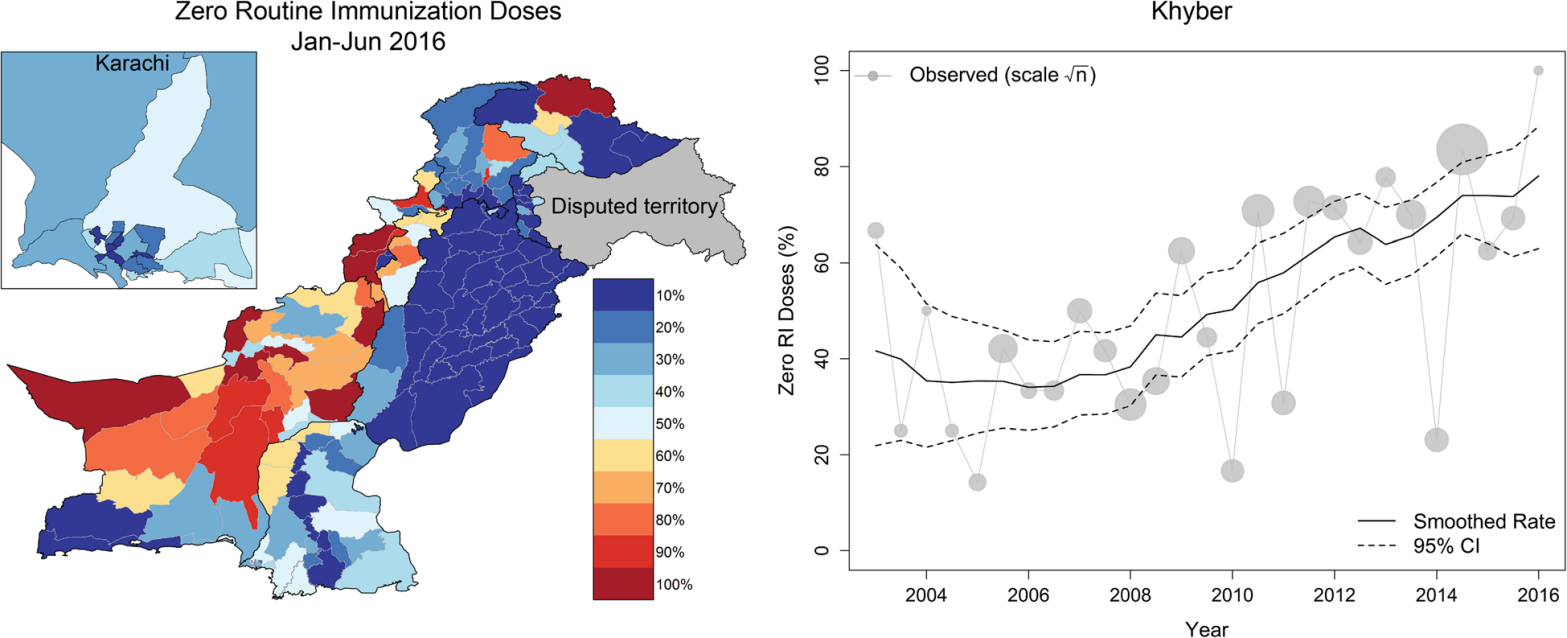
A map of smoothed estimates of zero routine immunization (RI) doses for January through June, 2016 (left) and an example of the smoothing models for observed zero RI rates for Khyber district in the Federally Administered Tribal Areas (FATA) of Pakistan from 2003 to 2016 (right)

**Fig. 2:**
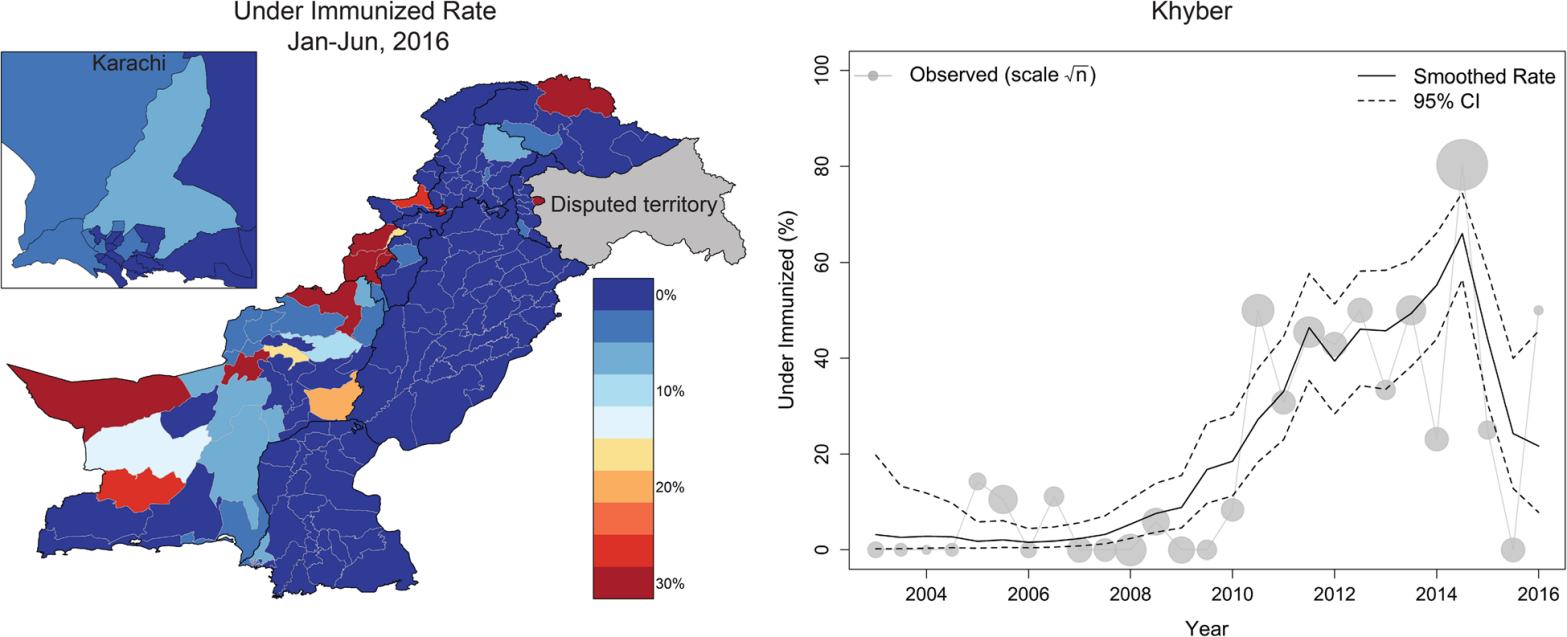
A map of smoothed estimates of underimmunized fraction for January through June, 2016 (left) and observed underimmunized fraction with smoothed estimates for Khyber district from 2003 to 2016 (right)

**Fig. 3:**
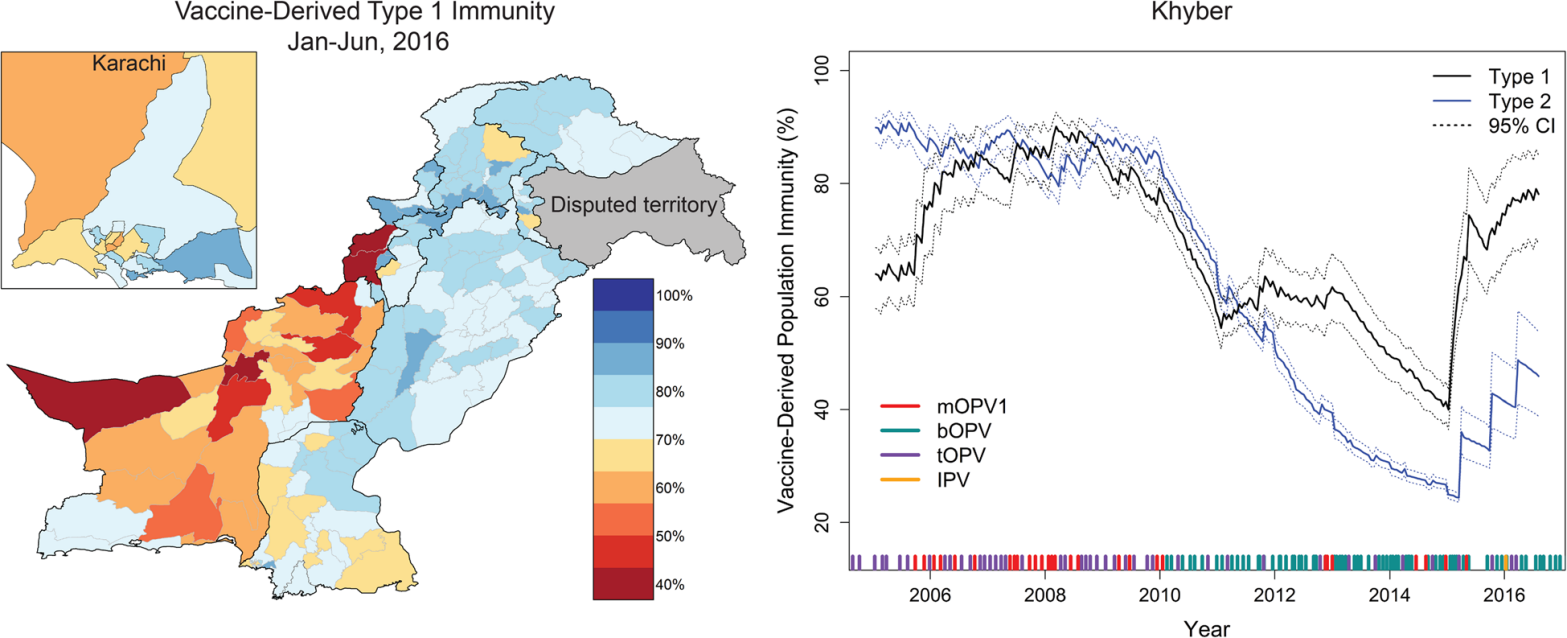
A map of the vaccine-derived population immunity for type 1 poliovirus estimated by the dynamic immunity model as of June 30, 2016 (left), and the dynamic immunity traces and 95% credible interval for immunity in Khyber with supplementary immunization activity (SIA) calendar from 2003 to 2016 (right). The dashed marks along the horizontal axis show the timing of the SIAs and the color represents the vaccine used in the SIA, where mOPV1 represents monovalent oral polio vaccine (OPV) for serotype 1, bOPV represents bivalent OPV for serotypes 1 and 3, tOPV represents trivalent OPV (serotypes 1-3), and IPV is the inactivated poliovirus vaccine

AFP surveillance in Pakistan collected data on 43,301 NPAFP cases between January 2003 and June 2016, with an average annual rate increasing from 4.3 to 11.4 NPAFP per 100,000 children under the age of 5 years from 2003 to 2016. Space–time smoothing models fit to the NPAFP vaccination dose history data indicated that zero dose RI and underimmunized rates (fewer than three doses) are highly heterogeneous across Pakistan (Figs. 1 and 2). Both zero dose RI and underimmunization rates were high in most of Punjab, Sindh, and KP provinces, and lowest in the western provinces, Balochistan and FATA. Zero dose RI rates were generally higher than underimmunization rates, suggesting apparent gaps in RI. Our dynamic immunity model indicates that immunity is spatially and temporally variable across Pakistan (Fig. 3) due to differences in SIA coverage and the vaccine efficacies for each serotype, which vary among the vaccines [13].

### Association between covariates and cases of WPV1

The model that achieved the lowest DIC for the Bernoulli and truncated Poisson portions of the hurdle model are shown in Table 1. Associations are in the expected directions with high season, higher underimmunization rates, recent cases, and recent neighboring cases being positively associated with at least one case (odds ratios above 1) and higher rates of population immunity being associated with a lower probability of a case. The number of cases given at least one case is best described by high season, underimmunization rate, zero-dose RI rate, recent cases, and recent neighboring cases. As expected, season, underimmunization rates, zero-dose RI rate, and recent neighboring cases are positively associated with cases (relative rates above 1).

**Table 1:** Posterior medians and 95% credible intervals of covariates selected for final risk model

	Bernoulli		Truncated Poisson	
Indicator	Odds ratio	95% CI	Relative rate	95% CI
Season (reference Jan–June)	3.13	(2.44–4.03)	2.52	(1.69–3.82)
Type 1 immunity (10% difference)	0.89	(0.81–0.99)		
Under immunization (10% difference)	1.12	(1.01–1.24)	1.28	(1.09–1.50)
Zero RI doses (10% difference)			1.24	(1.11–1.38)
Sqrt. recent cases	1.78	(1.44–2.19)	1.08	(0.90–1.30)
Sqrt. recent neighbor cases	1.18	(1.05–1.32)	1.31	(1.15–1.50)

Odds ratios are reported for the Bernoulli portion of the model and relative rates for the truncated Poisson

### Predictive performance

**Fig. 4:**
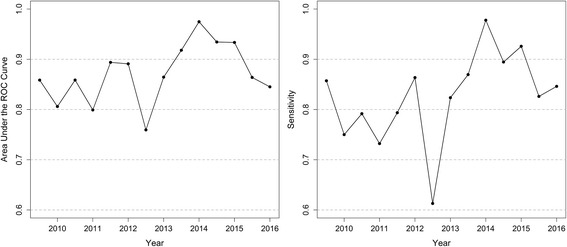
Area under the curve, the probability that a randomly selected district with a case will have a higher risk score than a randomly selected district without a case, for prediction of WPV1 cases by district as predicted based on model data from 2003 until 6 months prior to observed data (left) and sensitivity, or true positive rate, of a list containing the top 50 high risk districts for each time point (right)

The predictive accuracy of our selected model was high (Fig. 4). AUC values were consistently above 0.8 and often above 0.9. Over the past 3 years, the AUC ranged from 0.84 to 0.97, with a mean of 0.90. Furthermore, the sensitivity of a list made up of the districts with the 50 highest risk scores, that is the proportion of districts with a case that are ranked in the top 50 risk scores, ranged from 0.78 to 0.97 over the past 3 years. Both measures show good performance overall, aside from the second half of 2012, which had more districts in KP with cases than our model would have predicted.

### Predicted risk for WPV1

**Fig. 5:**
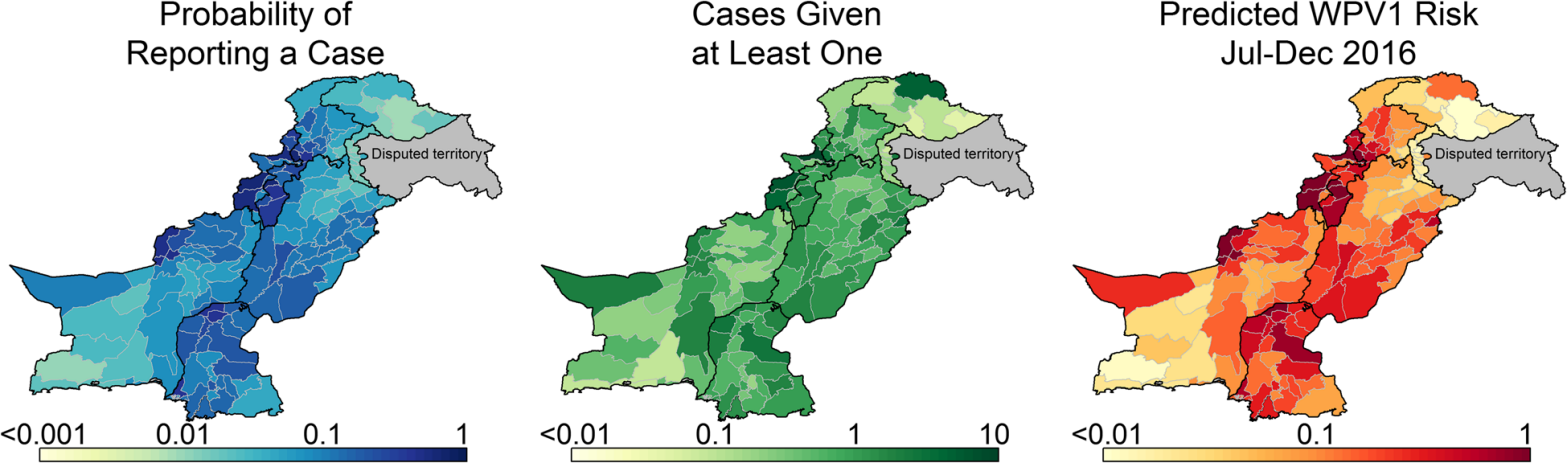
The probability of at least one case (left), expected number of cases given at least one case (center), and the overall risk score (expected number of cases) (right), for July through December, 2016

The probability of at least one WPV1 case is highest along the regions of KP, FATA, and Balochistan, which border Afghanistan, in northern Sindh, and near Karachi (Fig. 5). The expected number of cases given at least one case, which is driven primarily by population size and population immunity, is highest in areas with low immunity and high population. The final risk score, defined as the expected number of cases and described in Section 2 of Additional file 1, indicates that the highest risk areas are found along the border with Afghanistan in the Quetta block (the districts of Quetta, Pishin, and K. Abdullah), in Northern Sindh, and near Karachi.

### Programmatic impact of the risk model

**Fig. 6:**
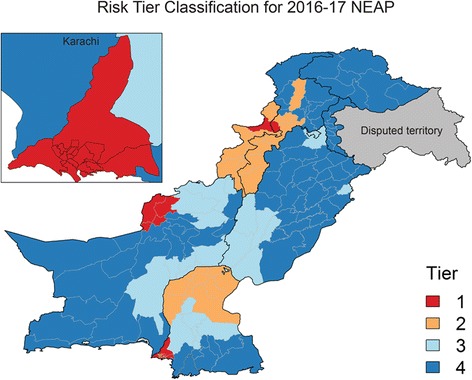
The final risk tier classification to be included in the National Emergency Action Plan for polio eradication in Pakistan for 2016–2017 with Karachi in the inset. This final list incorporates modeling output, genetic sequencing results, and local knowledge of access and security

The N-EOC, in coordination with Provincial EOCs, have used the outcomes of the model to assess the overall risk profile of the program. To operationalize the risk modeling results, a combination of the modeling output described here, genetic sequencing results (including virus isolated from environmental and healthy children stool samples), and local knowledge of access and security status of districts and Union Councils, as well as sociocultural links within Pakistan and across the border with Afghanistan, were used to classify the 155 districts into four tiers (Fig. 6). Tier 1 represents the top 11 districts considered ‘core reservoirs’ of poliovirus in Pakistan. Through genetic sequencing data, there is strong evidence of persistent circulation of local lineages of WPV for at least 2 years in the core reservoirs [6]. Tier 2 consists of the next 33 districts, considered high risk districts, while tier 3 districts are considered vulnerable districts. Tier 4 includes all other districts. Table 2 shows the programmatic implications of the tier classification on the immunization and operational strategies deployed.

**Table 2:** Programmatic implications of risk classifications of districts for July 2016 to June 2017

Tier	Number of districts	Target population (%)	Goal	Strategy
1	11	4,042,214 (11%)	Interrupt endemic and/or persistent local transmission using multiple strategies	NID + SNID + CBV in selected UCs + Priority 1 for combined bOPV/IPV SIA + RI service delivery support and other auxiliary support
2	33	5,746,129 (16%)	Interrupt transmission if transmission is ongoing, decrease vulnerability	NID + SNID + CBV in selected UCs + Priority 2 for bOPV/IPV SIA + RI service delivery support + other auxiliary support
3	24	7,246,474 (20%)	Decrease vulnerability	NID + SNID
4	87	19,638,741 (54%)	Maintain high population immunity	NID only

Tier classifications dictate inclusion in National Immunization Days (NIDs), Sub-National Immunization Days (SNIDs) and employment of community-based vaccination (CBV) strategies in selected Union Councils (UCs). Reproduction of Panel 1 in the National Emergency Action Plan ([6], p. 19)

*IPV* inactivated polio vaccine, *OPV* oral polio vaccine, *SIA* supplementary immunization activity, *RI* routine immunization

## Discussion

Our results indicate that seasonality, immunity, underimmunization rate, recent cases, and recent cases in a neighboring district are most predictive of at least one WPV1 case. The number of cases given at least one case is similarly predicted by seasonality, underimmunization rate, zero RI dose rate, recent cases, and recent neighboring cases. The point estimates of all associations were in the expected direction of lower immunity and dose history as well as recent cases being associated with increased risk of cases and larger outbreaks.

Our modeling efforts suggest that the large outbreaks in 2014 and the recent improvements over the past 2 years can be described by population immunity driven primarily by SIAs in FATA and KP provinces. For example, the dramatic decrease in serotypes 1 and 2 immunities in KP beginning in 2010 were partially the result of declining SIA coverage rates (despite the high frequency of campaigns). Furthermore, type 2 immunity declined due to infrequent trivalent OPV campaigns, whereas type 1 immunity declined because SIAs were primarily using bivalent and trivalent OPV, which has a lower efficacy for type 1 than monovalent OPV type 1 vaccine. Finally, improvements beginning in 2015 were due to improved vaccinator access driven by military intervention in FATA [33].

Based on the recommendations in the 2016–2017 NEAP, finalized in May of 2016, Tier 1–3 districts participated in four bOPV SIAs in addition to the five national bOPV SIAs that covered all districts. Additionally, Tier 1 districts scaled up the community-based vaccination strategy, which employs local individuals, primarily women who are thought to have better access to children within homes, as permanent vaccinators within their communities. In the 6-month period between July and December, 2016, only two of the 44 Tier 1 and 2 districts experienced cases (one each), which reflects well on the efforts focused on those districts. Two of the three other districts, which reported cases during this time frame, were classified as Tier 4 districts (four of five cases in Tier 4), although they were ranked in the top 30 per the risk model; the remaining Tier 4 district, in northern KP province, would be considered relatively surprising from a modeling and programmatic perspective. We emphasize that even Tier 4 districts received considerable programmatic attention, with five planned SIAs across 2016.

Our approach does have several limitations. We have developed a model on a 6-month time scale, which is programmatically relevant but does not align with the approximately 1 month infectious period estimated for poliovirus [34]. Additionally, we have modeled observed WPV1 paralytic cases that only represent approximately 0.5% of WPV1 infections [35]. This absence of cases could be misleading if circulation is silent due to surveillance failure, waned mucosal immunity among older children or adults [36, 37] or, as observed in Israel, high rates of humoral immunity due to exclusive inactivated polio vaccine use leading to low mucosal immunity [38]. Finally, areas that are not explained well by the covariates will have large residual risk captured by the random effects and, as these are invariant in time, we will likely overestimate risk in areas with a long history of WPV1 cases, despite improvements in indicators.

In 2009, the Pakistan program initiated environmental surveillance (ES) to compliment AFP surveillance. Since 2009, the program has grown from 47 samples across 6 sites to 648 samples across 62 sites covering 33 districts in 2016 [14, 39]. Due to the selective and expanding deployment of ES over time and the unique interpretation of ES positives, which signify at least one infection, ES data it is not easily included as a predictor in our currently modeling framework. Similarly, as sampling locations have been selected based on a history of persistent infection [39], we would expect positive samples to primarily reinforce the high-risk status of areas with a history of cases if included as an outcome. It is a limitation of our case-based statistical model that there is not a straightforward way to incorporate the ES data as either a predictor or an outcome. Alternative approaches, such as a transmission model that includes genetic information or a statistical model that incorporates the relative sensitivity of ES and AFP, may be better suited to incorporate the ES data. However, although it is not explicitly included in the risk model for WPV1 cases, ES is used extensively within the program to identify infected areas when transmission is low and is essential for assessing progress towards eradication.

Despite the limitations, our modeling approach provides a principled framework for ranking districts for risk classification that performs well as measured by AUC and sensitivity. In practice, the risk analysis generally identifies the same Tier 1 districts as the aggregation of programmatic knowledge and scientific intuition of the N-EOC members and the greatest impact of the modeling approach is the promotion of districts that appear susceptible based on immunity profile, but have not yet had WPV1 cases, to a lower (higher risk) tier. These promotions impacted the allocation of resources by deploying the community-based vaccination strategy in additional districts and broadening the geographic scope of the four sub-national SIAs. Additionally, the method provides a metric for quantifying the absolute risk and changes in risk over time, which is not always captured well by cases or intuition exclusively.

## Conclusion

This study serves as the first use of a spatial model for risk prediction and sub-national prioritization to aid in polio eradication in Pakistan. This risk modeling approach has been applied to the WPV1 case history and NPAFP dose histories to generate risk predictions and a ranked list of districts within Pakistan, which were subsequently used by the N-EOC to help assign districts to risk tiers as part of their NEAP for polio eradication. This approach will help maximize the impact of the resources available for polio eradication efforts in Pakistan.

## Additional file

Additional file 1: Supplemental Materials for “Spatial Model for Risk Prediction and Sub-National Prioritization to Aid Poliovirus Eradication in Pakistan”. (PDF 655 kb)

## Abbreviations

AFP: Acute flaccid paralysis
AUC: Area under the curve
DIC: Deviance information criteria
ES: Environmental surveillance
FATA: Federally Administered Tribal Areas
GPEI: Global Polio Eradication Initiative
INLA: Integrated Nested Laplace Approximation
KP: Khyber Pakhtunkhwa
NEAP: National Emergency Action Plan
N-EOC: National Emergency Operation Centre
NPAFP: Non-polio acute flaccid paralysis
OPV: Oral polio vaccine
RI: Routine immunization
ROC: Receiver operator characteristic
SIA: Supplementary immunization activity
WHO: World Health Organization
WPV1: Wild poliovirus type 1
