# Supplementary material for: Spatial model for risk prediction and sub-national prioritization to aid poliovirus eradication in Pakistan
Source: BMC Med. 2017 Oct 11;15:180. doi: 10.1186/s12916-017-0941-2 (PMC5635525; doi:10.1186/s12916-017-0941-2)
Supplement: Additional file 1: — Supplemental Materials for “Spatial Model for Risk Prediction and Sub-National Prioritization to Aid Poliovirus Eradication in Pakistan”. (PDF 655 kb) [file 12916_2017_941_MOESM1_ESM.pdf]

Supplemental Materials for "Spatial Model for Risk Prediction and Sub-National Prioritization to Aid Poliovirus Eradication in Pakistan"

1. Smoothing of vaccination rate covariates

The expected rate of acute flaccid paralysis (AFP) is 1 in 100,000 children under 5 years of age, thus the sample sizes from each district in a six-month period can be quite small, and range from 0 to 76 in our observation period. Figure 1 shows the in large variability in the observed rate of zero RI dose fraction over time in the Khyber and Quetta districts of Pakistan. It is demographically implausible that the true zero RI dose fraction could vary by as much as 40% in a six-month time period. Thus, instead of including the observed under immunized fractions and zero routine immunization fractions in our models we used a space-time smoothing model to estimate an underlying rate from which these observations were drawn

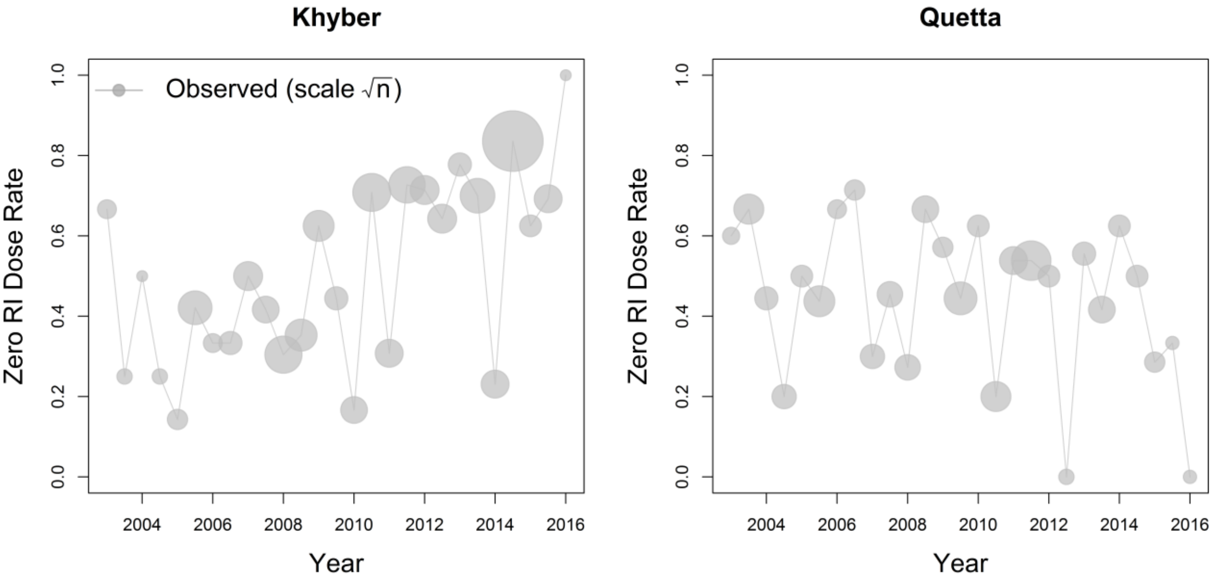

Figure S.1: Observed zero routine immunization rates by six-month time periods in Khyber (left) and Quetta (right) districts of Pakistan from 2003-2016. Point size is scaled by the square root of the number of NP-AFP observations.

To estimate the underlying rates we considered the hierarchical Bayesian space-time smoothing models.[1] In the first stage, for each indicator we assume the count in district i and time period t (X_{it}) is distributed $X_{it} \sim Bin(m_{it}, q_{it})$ where m_{it} is the number of NP-AFP cases and q_{it} and is the underlying rate of interest. We consider the logit linear model

$$\text{logit}(q_{it}) = \mu + \theta_i + \phi_i + \alpha_t + \gamma_t + \delta_{it}$$

where μ is the overall risk level, θ_i is a spatially structured effect of district i, ϕ_i independent effect of time, α_t is the temporally structured, γ_t is the independent effect of time t, and δ_{it} is the space-time interaction.

At the second stage of the hierarchical model, we assign priors to the random effects. The independent effects are assigned the priors $\phi_i|\sigma_\phi^2 \sim N(0, \sigma_\phi^2)$ for $i=1,\ldots,I$ and $\gamma_t|\sigma_\gamma^2 \sim N(0, \sigma_\gamma^2)$ for $t=1,\ldots,T$. The temporally structured effect is assigned a second order random walk prior where $\alpha_t|\alpha_{-t}, \sigma_\alpha^2 \sim N(4(\alpha_{t+1} + \alpha_{t-1})/6 - (\alpha_{t+2} + \alpha_{t-2})/6, \sigma_\alpha^2/6)$. The spatially structured effect is assigned the intrinsic conditional autoregressive prior (ICAR)[2] where $\theta_i|\theta_{-i}, \sigma_\theta^2 \sim N(\sum_{j\sim i}\theta_j/m_i, \sigma_\theta^2/m_i)$, where $j \sim i$ denotes the districts that have a shared boundary with district i, θ_{-i} is the vector of θs excluding θ_i, and m_i is the number of districts that share a boundary with district i. Additional details about the temporally and spatially structured priors can be found in *Gaussian Markov Random Fields*.[3] The inverse of all variance parameters were assigned gamma distribution priors with shape 1 and scale 0.00005.

We considered three variations of this space-time model. In the first we consider no space-time interaction (no δ_{it} parameter). In the second model an unstructured prior is assigned $\delta_{it} | \sim N(\mathbf{0}, \sigma_\delta^2)$. In the final model we considered a second order random walk for each district $\delta_{it} \sim N(4(\delta_{i,t+1} + \delta_{i,t-1})/6 - (\delta_{i,t+2} + \delta_{i,t-2})/6, \sigma_\delta^2/6)$. All of the random effect variances were assigned inverse Gamma priors. Models were implemented using the Integrated Nested Laplace Approximation (INLA) in the computing environment R (R Core Team, 2015) as described by Schrödle and Held (2011).

The estimated under immunization rates and zero routine immunization dose rates based on the model with no space-time interaction, an independent interaction, and the temporally structured interaction are shown in Figures 2 and 3, respectively. In both Figures 2 and 3, we see that without including a space-time interaction we do not allow different districts to have different temporal trends, which is an unrealistic restriction, with the estimates for Khyber looking especially poor. Additionally, the independent space-time interaction appears to be too flexible and does not impose sufficient smoothing. However, the model with the temporally structured interaction, which corresponds to a type II space-time interaction in Schrödle and Held (2011), provides reasonable trends for both rates and was selected for smoothing the covariates.

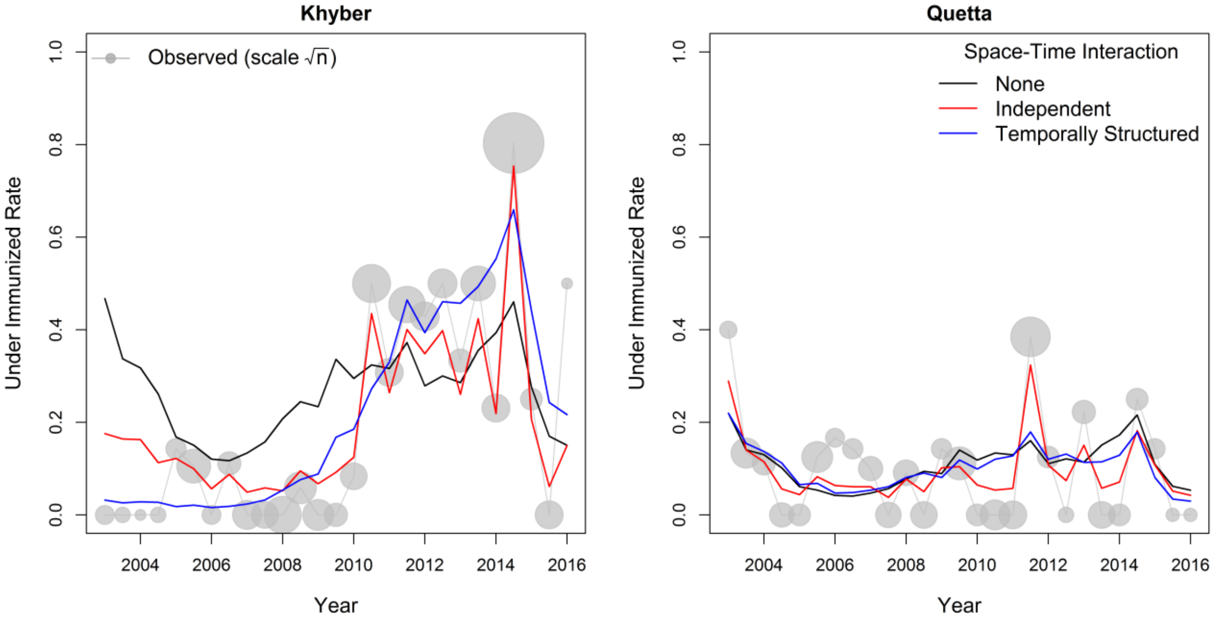

Figure S.2: Observed under immunized (less than three OPV doses) rate and smoothed values based on models with independent, temporally structured, or no space-time interaction in Khyber and Quetta districts of Pakistan.

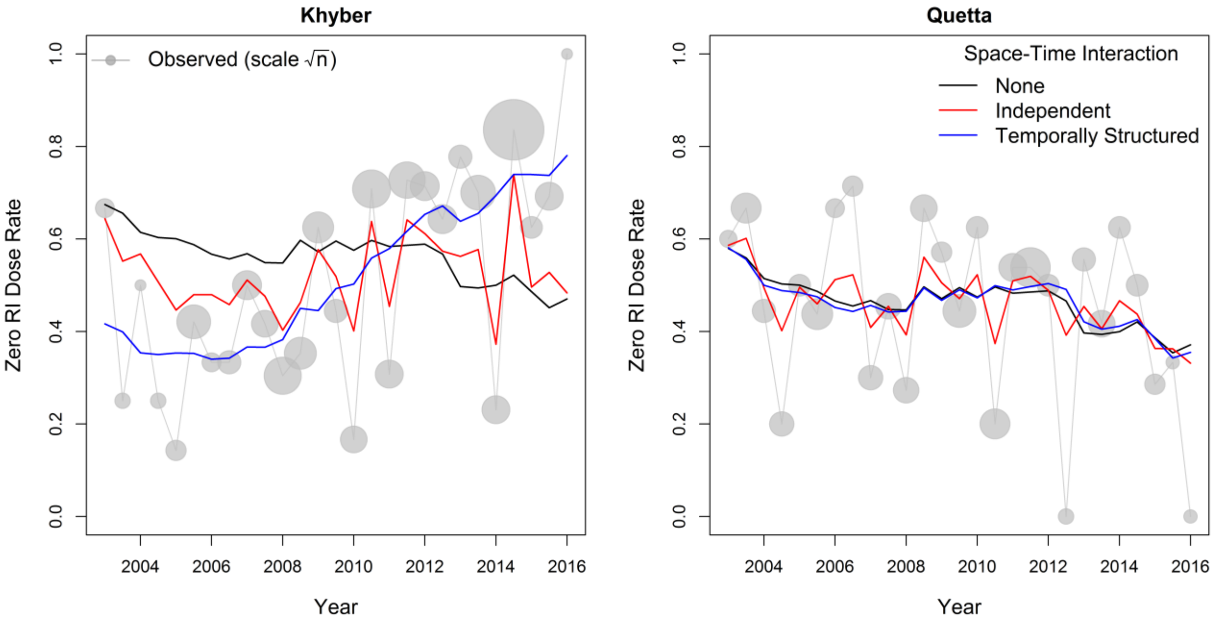

Figure S.3: Observed zero routine immunization dose rate and smoothed values based on models with independent, temporally structured, or no space-time interaction in Khyber and Quetta districts of Pakistan.

2. Poisson Hurdle Model

To model the risk of wild poliovirus serotype one (WPV1) in districts of Pakistan we implemented a spatial Poisson hurdle model[6] similar to the model that was used to model WPV1 and WPV3 in Nigeria by Upfill-Brown et al. (2014). The spatial Poisson hurdle model is a two-part model that jointly models the probability of at least one WPV1 and the number of WPV1 given at least one.

Formally, if we assume y_{it} is the number of confirmed WPV1 cases in district i at time t, for $i = 1, \ldots, I$ and $t = 1, \ldots, T$, and z_{it} is an indicator of $y_{it} > 0$, then we model $z_{it}|p_{it} \sim \text{Bern}(p_{it})$ with

$$\text{logit}(p_{it}) = \eta_1 + \boldsymbol{\beta}_1 \boldsymbol{X}_{i,t-1,1} + u_{i1} + v_{i1}$$

and for $z_{it} = 1$ we model $y_{it}|\lambda_{it} \sim \text{Truncated Poisson}(\lambda_{it})$ with

$$\log(\lambda_{it}) = \eta_2 + \boldsymbol{\beta}_2 \boldsymbol{X}_{i,t-1,2} + u_{i2} + v_{i2} + \epsilon_{it} + \log(N_{it})$$

where η_1 and η_2 are the intercepts, $\boldsymbol{\beta}_1$ and $\boldsymbol{\beta}_2$ are the coefficients for the indicators, u_{i1} and u_{i2} represent independent district effects, v_{i1} and v_{i2} represent spatially structured district effects, ϵ_{it} are observation level random effects to account for overdispersion, and N_{it} is the population of district i at time t. The $\boldsymbol{X}_{i,t-1,1}$ and $\boldsymbol{X}_{i,t-1,2}$ represent the covariates, such as smoothed zero routine immunization dose rate, from district i at time $t-1$. In our application of the model this represents a 6-month lag.

The truncated Poisson distribution takes the form

$$P(Y_{it} = k) = p_{it} \frac{\lambda_{it} e^{-\lambda_{it}}}{k! \, \{1 - e^{-\lambda_{it}}\}}, \quad k = 1, \ldots, \infty,$$

where k is the number of WPV1. The expected number of WPV1 for the next time period $t+1$ takes the form

$$E[Y_{i,t+1}] = p_{i,t+1} \cdot \frac{\lambda_{i,t+1}}{1 - e^{-\lambda_{i,t+1}}}$$

and is used as the "risk score" for ranking the districts.

In the hierarchical Bayesian setting we also need to assign prior distributions to each parameter. Flat priors are assigned to η_1 and η_2. Diffuse normal priors are assigned to the covariate effects $\boldsymbol{\beta}_1$ and $\boldsymbol{\beta}_2$. The district-level random effects are assigned a bivariate Normal prior, $[u_{i1}, u_{i2}]^T|\Sigma \sim N_2(\mathbf{0}, \Sigma)$ with $\Sigma = \begin{bmatrix} \sigma_{u1}^2 & \rho \\ \rho & \sigma_{u2}^2 \end{bmatrix}$ and the spatially structured random effects v_{i1} and v_{i2} are assigned ICAR priors. Finally, the Wishart is assigned to Σ^{-1} with 4 degrees of freedom and a scale matrix with ones on the diagonal entries and zeros on the off-diagonal entries and gamma distributions with shape 1 and scale 0.00005 are assigned to the inverse of the variance components of the ICAR priors and the observation level random effect.

Bibliography

1 Knorr-Held L. Bayesian Modelling of Inseperable Space-time Variation in Disease Risk. *Stat Med* 2000; **19**: 2555–67.

2 Besag J. Spatial interaction and the statistical analysis of lattice systems. *J R Stat Soc Ser B* 1974; **36**: 192–236.

3 Rue H, Held L. Gaussian Markov Random Fields: Theory and Application. Boca Raton: Chapman and Hall/CRC Press, 2005.

4 R Core Development Team. R: a language and environment for statistical computing, 3.2.1. Doc. Free. available internet http//www. r-project. org. 2016. DOI:10.1017/CBO9781107415324.004.

5 Schrödle B, Held L. Spatio-temporal disease mapping using INLA. *Environmetrics* 2011; **22**: 725–34.

6 Neelon B, Ghosh P, Loebs PF. A spatial Poisson hurdle model for exploring geographic variation in emergency department visits. *J R Stat Soc Ser A (Statistics Soc* 2013; **176**: 389–413.

7 Upfill-Brown AM, Lyons HM, Pate MA, *et al.* Predictive spatial risk model of poliovirus to aid prioritization and hasten eradication in Nigeria. *BMC Med* 2014; **12**: 92.
